# Right Ventricular Function in Acute Respiratory Distress Syndrome: Impact on Outcome, Respiratory Strategy and Use of Veno-Venous Extracorporeal Membrane Oxygenation

**DOI:** 10.3389/fphys.2021.797252

**Published:** 2022-01-14

**Authors:** Matthieu Petit, Edouard Jullien, Antoine Vieillard-Baron

**Affiliations:** ^1^Medical Intensive Care Unit, University Hospital Ambroise Paré, APHP, Boulogne-Billancourt, France; ^2^UFR des Sciences de la Santé Simone Veil, Université Paris-Saclay, Montigny-le-Bretonneux, France

**Keywords:** ARDS, right ventricle, VV ECMO, echocardiography, acute cor pulmonale (ACP)

## Abstract

Acute respiratory distress syndrome (ARDS) is characterized by protein-rich alveolar edema, reduced lung compliance and severe hypoxemia. Despite some evidence of improvements in mortality over recent decades, ARDS remains a major public health problem with 30% 28-day mortality in recent cohorts. Pulmonary vascular dysfunction is one of the pivot points of the pathophysiology of ARDS, resulting in a certain degree of pulmonary hypertension, higher levels of which are associated with morbidity and mortality. Pulmonary hypertension develops as a result of endothelial dysfunction, pulmonary vascular occlusion, increased vascular tone, extrinsic vessel occlusion, and vascular remodeling. This increase in right ventricular (RV) afterload causes uncoupling between the pulmonary circulation and RV function. Without any contractile reserve, the right ventricle has no adaptive reserve mechanism other than dilatation, which is responsible for left ventricular compression, leading to circulatory failure and worsening of oxygen delivery. This state, also called severe acute cor pulmonale (ACP), is responsible for excess mortality. Strategies designed to protect the pulmonary circulation and the right ventricle in ARDS should be the cornerstones of the care and support of patients with the severest disease, in order to improve prognosis, pending stronger evidence. Acute cor pulmonale is associated with higher driving pressure (≥18 cmH_2_O), hypercapnia (PaCO_2_ ≥ 48 mmHg), and hypoxemia (PaO_2_/FiO_2_ < 150 mmHg). RV protection should focus on these three preventable factors identified in the last decade. Prone positioning, the setting of positive end-expiratory pressure, and inhaled nitric oxide (INO) can also unload the right ventricle, restore better coupling between the right ventricle and the pulmonary circulation, and correct circulatory failure. When all these strategies are insufficient, extracorporeal membrane oxygenation (ECMO), which improves decarboxylation and oxygenation and enables ultra-protective ventilation by decreasing driving pressure, should be discussed in seeking better control of RV afterload. This review reports the pathophysiology of pulmonary hypertension in ARDS, describes right heart function, and proposes an RV protective approach, ranging from ventilatory settings and prone positioning to INO and selection of patients potentially eligible for veno-venous extracorporeal membrane oxygenation (VV ECMO).

## Introduction

Acute respiratory distress syndrome (ARDS) is characterized by protein-rich alveolar edema, reduced lung compliance and severe hypoxemia (Thompson et al., 2017). Despite some evidence of improvements in mortality over recent decades (Brun-Buisson et al., 2004; Phua et al., 2009) due to better understanding of its pathophysiology and routine application of protective mechanical ventilation, ARDS remains a major public health problem with an approximately 30% 28-day mortality in recent cohorts (Bellani et al., 2016; Combes et al., 2018; Constantin et al., 2019). Pulmonary vascular dysfunction (Snow et al., 1982; Price et al., 2012) is one of the pivot points of the pathophysiology, resulting in a certain degree of pulmonary hypertension, higher levels of which are associated with morbidity and mortality (Bull et al., 2010). The hemodynamic consequences of such remodeling of the pulmonary circulation has led clinicians to pay attention to the right ventricle as the deleterious impact of right ventricular (RV) failure on prognosis is well demonstrated (Mekontso Dessap et al., 2016).

This review reports the pathophysiology of pulmonary hypertension and RV injury, describes RV function, and explains the interest of proposing a RV protective approach to manage ARDS patients, ranging from ventilatory settings and prone positioning to nitric oxide (NO) inhalation and selection of patients potentially eligible for veno-venous extracorporeal membrane oxygenation (VV ECMO) in this context. A few specificities of ARDS-related COVID-19, if any, will be mentioned.

## Pathophysiology of Right Ventricular Injury in Acute Respiratory Distress Syndrome

### Right Ventricular Physiology

The right ventricle is composed of the filling chamber and the outflow chamber. Under normal conditions, the right ventricle ejects the blood into the pulmonary circulation, a system of low resistance and high compliance. In contrast to the left ventricle, its isovolumetric contraction pressure is very low and its isovolumetric relaxation is insignificant (Redington et al., 1990): it acts as a passive conduit. This is why its systolic function is sensitive to any increase in pulmonary vascular resistance (PVR) with no adaptation reserve, leading to dysfunction and ultimately to failure. However, the right ventricle is able to adapt to a certain degree of pulmonary hypertension by dilating, due to its high diastolic compliance (Laks et al., 1967).

### Pulmonary Vascular Dysfunction

ARDS is characterized by acute onset hypoxemia (ARDS Definition Task Force et al., 2012) with increased pulmonary vascular permeability, leading to non-cardiogenic pulmonary edema (Ashbaugh et al., 1967). Along with alveolar damage, ARDS directly causes injury to the pulmonary circulation, through several pathophysiological mechanisms, involving endothelial dysfunction, distal pulmonary vascular occlusion at the level of the capillaries, pulmonary vasoconstriction, extrinsic vessel occlusion by alveoli distension and ultimately vascular remodeling (Price et al., 2012). All of these phenomena lead to elevation of PVR, pre-capillary pulmonary hypertension and increased RV afterload.

In COVID-19, a certain “protection” of the pulmonary circulation could occur with first the development of pulmonary angiogenesis (Ackermann et al., 2020) and second the virtual absence of hypoxic pulmonary vasoconstriction (Archer et al., 2020). Conversely, proximal obstruction of the pulmonary circulation has been reported to be frequent.

### Focus on the Effect of Mechanical Ventilation

**FIGURE 1:**
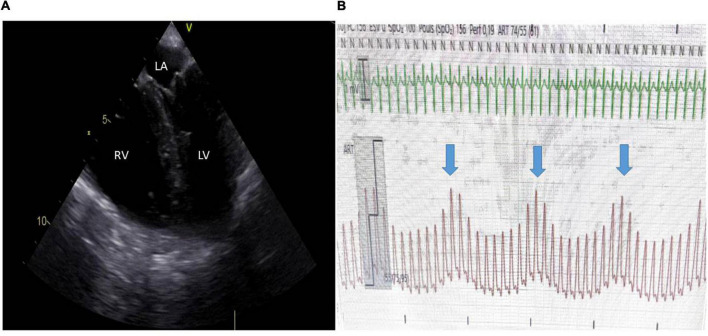
Acute cor pulmonale in a patient ventilated for ARDS and in shock and completely adapted to the respirator. **(A)** A mid-esophageal 4-chamber view demonstrated severe RV dilatation with paradoxical septal motion. **(B)** Invasive low blood pressure with significant pulse pressure variation (blue arrows indicate insufflation) through a radial catheter. Central venous pressure was also elevated. LV, left ventricle; LA, left atrium; RV, right ventricle.

Inadequate mechanical ventilation may have a deleterious effect on RV function. During spontaneous breathing in a healthy subject, RV function is optimal with adequate venous return due to negative pleural pressure (Guyton et al., 1957), and RV afterload is limited because of a low transpulmonary pressure (TPP) as lung compliance is normal. In ARDS, a situation where lung compliance is decreased, positive pressure ventilation induces increased TPP at least during tidal ventilation and sometimes during expiration in the case when too high a positive end-expiratory pressure (PEEP) is applied. As a consequence, the pulmonary capillaries are stretched and their caliber reduced, resulting in an increase in PVR (Whittenberger et al., 1960; West et al., 1964). Cyclic increase in PVR during tidal ventilation is responsible for cyclic changes in RV afterload, and then in RV outflow (Vieillard-Baron et al., 1999) eventually leading to pulse pressure variations (Figure 1). At the same time, ventilator settings may indirectly impact the pulmonary circulation through changes in PaO_2_ and PaCO_2_, both of which strongly mediate pulmonary vasoconstriction (Yamamoto et al., 2001).

It was suggested at least at the beginning of the COVID-19 pandemic that lung compliance was less decreased than in classical ARDS (Gattinoni et al., 2020), thus potentially inducing less interaction with the pulmonary circulation. This is, however, still questionable.

### Right Ventricular Failure and Acute Cor Pulmonale

Acute cor pulmonale (ACP) is the last stage of the uncoupling between the right ventricle and the pulmonary circulation. It could be understood, especially in its most severe form, as an RV failure state. RV afterload is suddenly increased, and RV ejection is impaired. In consequence, the right ventricle increases in size. This RV dilatation participates in circulatory failure by compressing the left ventricle (LV) (Scharf et al., 1979). Moreover, in normal conditions, RV and LV systoles occur simultaneously, with the right and left ventricles starting and ending contraction almost at the same time. When RV systole is overloaded, RV contraction is prolonged, so that the right ventricle continues to push after the left ventricle has ended, and the pressure in the RV cavity is then higher than the pressure in the LV cavity during a short instant (Elzinga et al., 1980). This explains the paradoxical septal motion observed in ACP (Figure 1A).

## Acute Cor Pulmonale: Incidence, Risk Factors, and Impact on Outcome

Prior to the widespread use of protective ventilation, ACP was reported in almost 60% of patients (Jardin et al., 1985). However, all patients were ventilated with high tidal volume and plateau pressure (Pplat) and all patients with severe RV dilatation finally died (Jardin et al., 1985). Since the era of protective ventilation, the incidence of ACP has declined to between 20 and 30% (Vieillard-Baron et al., 2001; Page et al., 2003; Mekontso Dessap et al., 2010, 2016), but may still be as high as 50% in the most severe ARDS (Vieillard-Baron et al., 2007). This leads physicians to take into consideration RV function in management strategies of patients with moderate to severe ARDS.

We still lack convincing data on the incidence of RV failure/ACP in ARDS related to COVID-19. One preliminary study in a very small series of patients reported an incidence of 17% (Evrard et al., 2020). Other studies not only including critically ill patients reported an RV dilatation in 35% of cases (Dweck et al., 2020) or an impact of RV dilatation on ICU transfer or death (Soulat-Dufour et al., 2021). In 90 COVID-19 patients, Bleakley et al. (2021) reported that radial RV dysfunction was common, while the longitudinal function was relatively spared. Micro-occlusive vasculopathy was also reported in COVID-19 by dual energy CT and was more clearly associated with RV dysfunction than the pulmonary embolism obstruction score (Ridge et al., 2020).

The largest study reporting risk factors for developing ACP was performed in 752 patients with moderate to severe ARDS submitted to protective ventilation (Mekontso Dessap et al., 2016). Driving pressure ≥ 18 cmH_2_O, PaCO_2_ ≥ 48 mmHg, PaO_2_/FiO_2_ < 150 mmHg and pneumonia as causes of ARDS identified patients at risk of ACP. Incidence of ACP ranged from less than 10% when only one risk factor was present to close to 60% with 3–4 risk factors (Mekontso Dessap et al., 2016). Interestingly, neither Pplat nor PEEP was reported as a potential risk factor. An explanation could be that a low PEEP was homogenously applied (mean 8 cmH_2_O) and Pplat was maintained below 27 cmH_2_O in most patients. In other conditions, they both may affect pulmonary circulation and RV function. A high Pplat is associated with RV failure, especially when it reflects high TPP (Vieillard-Baron et al., 1999). The “safe Pplat” for the right ventricle was suggested to be below 27 cmH_2_O (Jardin and Vieillard-Baron, 2007). Pplat is not always a surrogate of lung stress, because it reflects the compliance of the respiratory system (Gattinoni et al., 2004; Chiumello et al., 2008) and chest wall compliance must be taken into account, especially in obese patients. Monitoring of pleural pressure with an esophageal balloon could be of value in these patients, while data are missing. This could also be a specificity of COVID-19 patients who could tolerate higher Pplat, as many patients are obese and the association between Pplat and outcome in this subpopulation is unclear (De Jong et al., 2018).

The potential effect of PEEP on RV function is more questionable. Because of the opposite effect of lung distension on intra- and extra-alveolar pulmonary blood vessels, the relationship between lung distension and PVR is U-shaped (Whittenberger et al., 1960). Thus, the choice of the level of PEEP set by the clinician can directly affect the RV afterload because poor lung aeration on one side and alveolar overdistension on the other side can both raise PVR. In an experimental study, RV function was impaired when the lung was de-recruited and normalized after re-aeration (Duggan et al., 2003). As a matter of fact, lung CT-scan has shown a low amount of potentially recruitable lung (and so a high potential for overdistension) in most ARDS patients (Gattinoni et al., 2006) and a high PEEP was shown to induce hemodynamic instability more frequently in a randomized controlled trial, while no information was given on RV function, which was associated with worse outcome [Writing Group for the Alveolar Recruitment for Acute Respiratory Distress Syndrome Trial (ART) Investigators, 2017]. Despite a strict limitation of Pplat, a PEEP of 15 cmH_2_O produced a significant increase in PVR associated with a decrease in cardiac output (Schmitt et al., 2001). The reasonable goal of PEEP is then to reach a balance between enough recruitment and no or minimal overdistension. In other words, the goal is to set the best PEEP to recruit the zones of the collapsed lung, which typically characterize ARDS (Puybasset et al., 2000), without inducing alveolar dead space. Nowadays, no definitive manner to determine the best PEEP is available, but RV function evaluation can be used as a monitoring parameter to avoid PEEP resulting in too much overdistension.

As briefly discussed above, hypercapnia induces pulmonary vasoconstriction (Kiely et al., 1996). Hypercapnia is the consequence of respiratory strategy, i.e., protective ventilation designed to reduce ventilator-induced lung injury, but which also reflects the severity of ARDS (Nuckton et al., 2002).

Finally, one of the prognostic factors in ARDS is hemodynamic instability. And RV failure is one of its mechanisms. While still debatable, many arguments suggest that pulmonary vascular dysfunction and RV failure/ACP could thus have a negative impact on in-hospital mortality (Bull et al., 2010; Mekontso Dessap et al., 2016). This leads to discussion of the potential interest of an RV protective ventilation strategy.

## Evaluation of Right Ventricular Function at the Bedside

Historically, a pulmonary arterial catheter has been used to evaluate RV function at the bedside. Some of the key elements of monitoring proposed were as follows: low cardiac output, right atrial pressure (RAP) higher than pulmonary artery occlusion pressure (PAOP), and pulmonary hypertension. Recently, the so-called transpulmonary gradient, i.e., the difference between mean pulmonary artery pressure and PAOP, was reported to be frequently abnormally increased and associated with outcome (Bull et al., 2010). However, use of a pulmonary arterial catheter has progressively declined and critical care echocardiography has been progressively implemented and performed in ARDS (Dres et al., 2018). Echocardiographic definition of RV injury is still challenging but in ARDS ACP or severe RV dilatation accurately reflects RV failure, especially when RAP is elevated (Vieillard-Baron et al., 2018). It is recommended by experts in the field to monitor RAP and invasive blood pressure and to perform echocardiography (Vieillard-Baron et al., 2016).

## Right Ventricular Protective Strategy

**FIGURE 2:**
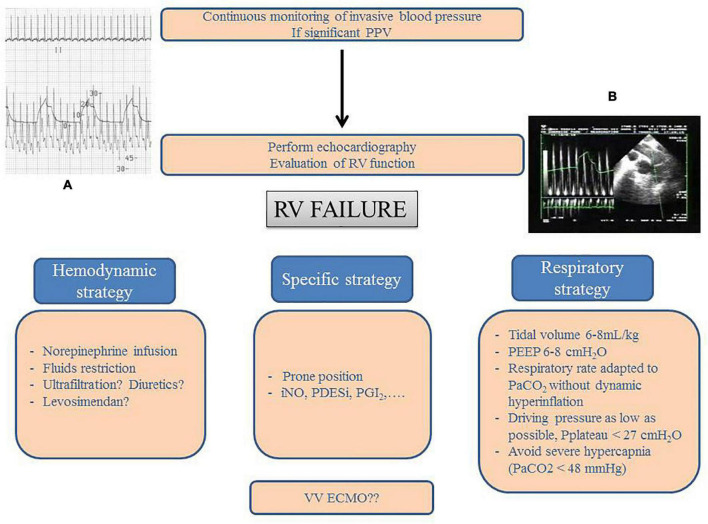
Right ventricle protective strategy. Principles for detection and management of right ventricular (RV) failure in patients with ARDS. This should combine invasive blood pressure monitoring and echocardiography. In the case of significant pulse pressure variation (PPV, Panel **A**), critical care echocardiography must be performed. It usually demonstrates cyclic decrease in RV outflow at each insufflation (Panel **B** on an upper esophageal view with pulsed wave Doppler into the main pulmonary artery) with either isolated RV dilatation or acute cor pulmonale. Management is based on three different strategies: hemodynamic, respiratory, and specific. VV ECMO can be considered in the case of persistent RV failure. PEEP, positive end-expiratory pressure; iNO, inhaled nitric oxide; PDESi, phosphodiesterase type 5 inhibitor; PGI_2_, prostaglandin I_2_; VV ECMO, veno-venous extracorporeal membrane oxygenation.

The main “rules” for protecting the RV in ARDS, by means of avoiding or correcting RV failure, are reported in Figure 2. While in our usual practice, we apply systematic daily evaluation of RV function by echocardiography in ARDS patient, Figure 2 also allows to reemphasize that when echocardiography is not so easily available, pulse pressure variation should be understood as a marker of a deleterious interaction between the RV and the ventilator and then requires further hemodynamic evaluation by echocardiography.

### Ventilatory Strategy

As largely discussed above in the physiological rationale, Pplat should be maintained below 27 cmH_2_O, and permissive hypercapnia should be limited by careful increase in respiratory rate and by replacing the heat and moisture exchanger by a heated humidifier. Oxygenation should also be increased without too much PEEP (Vieillard-Baron et al., 2013), with a view to optimizing arterial oxygen delivery rather than PaO_2_/FiO_2_. Indeed, it has long been known that increased PEEP may improve oxygenation but reduce oxygen delivery because of its potential negative hemodynamic effect (Kumar et al., 1970).

### Prone Positioning

In the most severe ARDS, it is unlikely that all of the predefined goals of an RV protective approach will be reached. In this situation, prone positioning has been reported to efficiently unload the right ventricle (Vieillard-Baron et al., 2007). It improves oxygenation without increasing PEEP and decreases hypercapnia and Pplat due to lung recruitment of the dependent areas of the lung without overdistension of the non-dependent areas (Guérin et al., 2020), rendering lung ventilation more homogeneous.

To optimize hemodynamic improvement, prone positioning should be performed without chest support, which may be responsible for a decrease in systemic venous return and cardiac output due to excessive elevation of intra-thoracic pressure (Chiumello et al., 2006; Brown et al., 2013).

### Hemodynamic Support and Nitric Oxide Inhalation

When RV failure induces circulatory failure, hemodynamic support is based on two key principles: (i) strongly limit fluid expansion and (ii) restore blood pressure.

Fluid expansion may by itself induce RV failure (Patterson and Starling, 1914) and increase RAP and systemic congestion, leading to acute kidney injury (Chen et al., 2016, 2017). Moreover, it is very unlikely that fluid expansion increases cardiac output, even though significant pulse pressure variation, a marker of LV preload dependency, is observed (Vieillard-Baron et al., 2016; Figure 1). Correction of blood pressure by infusion of catecholamines helps improve RV function. In other experimental models of RV failure-related pulmonary circulation obstruction, norepinephrine decreases RV wall stress and RV end-diastolic pressure and improves RV stroke volume, unlike fluid expansion (Ghignone et al., 1984). One of supposed mechanisms is that norepinephrine corrects the functional RV ischemia induced by high RV wall stress combined with low blood pressure (Guyton et al., 1954; Vlahakes et al., 1981). The same observation was made in lung injury (Prewitt and Ghignone, 1983; Vieillard-Baron et al., 2003). In the case of associated LV systolic dysfunction, as observed in ARDS-related septic shock, dobutamine acting on both ventricles may be preferred, though there is no study supporting this approach.

Levosimendan is another inotropic drug called inodilator, acting *via* troponin C calcium binding. It was proposed when there is uncoupling between the right ventricle and the pulmonary circulation. This is strongly physiologically based in ARDS, but only one pilot study suggests an improvement in RV performance in ARDS patients (Morelli et al., 2006). Due to the potential side effects of levosimendan, more data are needed before making any recommendation.

Nitric oxide inhalation has nowadays been abandoned in ARDS after studies and meta-analyzes reported no beneficial effect on outcome (Gebistorf et al., 2016). However, the use of NO for a hemodynamic indication in a subgroup of patients with refractory RV failure despite respiratory optimization has never been evaluated. NO inhalation has been found to significantly decrease RV afterload in ARDS, especially in the case of hypercapnia (Puybasset et al., 2000).

In COVID-19-related ARDS, NO inhalation has been poorly studied, but the rationale is not strongly favored due to the virtual absence of hypoxic vasoconstriction. A few studies have reported an improvement in oxygenation (Longobardo et al., 2021; Robba et al., 2021), especially when cardiac biomarkers were elevated (Garfield et al., 2021), but no association was reported with RV function improvement (Bagate et al., 2020). However, the subgroup of patients with RV failure was not specially studied. In the absence of clear evidence, NO inhalation could be initiate when RV failure is persistent despite RV protective ventilator strategy or when prone position is contraindicated.

### Veno-Venous Extracorporeal Membrane Oxygenation

The EOLIA trial suggested that ECMO could be effective in some of the most severe cases of ARDS, but failed to demonstrate a 20% increase in survival (Combes et al., 2018). One of the reasons, despite the non-negligible proportion of crossover between control patients and ECMO patients, could be that criteria for selecting eligible patients were mainly based on blood gas analysis, as proposed by the Berlin classification (Ferguson et al., 2012). By easily controlling blood oxygenation and decarboxylation (Schmidt et al., 2013), VV ECMO suppresses two of the major factors of raised PVR in ARDS and could then be sufficient to unload the right ventricle without the use of veno-arterial (VA) ECMO (Miranda et al., 2015). VV ECMO could also promote ultra-protective ventilation which could benefit the right ventricle by a more pronounced reduction of Pplat and driving pressure (Schmidt et al., 2019). Considering the inevitable complications of VV ECMO, including severe bleeding (Combes et al., 2018), better selection of patients is essential. How this subgroup of patients with severe ARDS and RV failure could be considered as the ideal target remains to be evaluated, while a recent pilot study showed in a non-selected echocardiographic cohort of severe ARDS patients fulfilling the EOLIA criteria that driving pressure and RV failure were the only two factors associated with ICU mortality, in contrast to classical severity markers in ARDS (Petit et al., 2021). Pre-ECMO implantation RV dysfunction is not rare and has an approximately 30% incidence of RV dilatation (Lazzeri et al., 2018).

Another potential technique to support the right ventricle is extracorporeal CO_2_ removal. Data are too scarce for discussion of any recommendation (Papazian et al., 2019), but an experimental study in a porcine model of ARDS showed that CO_2_ removal is able to decrease RV afterload and to improve coupling between the right ventricle and the pulmonary circulation (Morimont et al., 2015). Such a technique could be efficient and valuable in protecting the right ventricle in the case of severe ARDS with significant hypercapnia and RV failure despite application of an RV protective strategy, but probably does not promote ultraprotective ventilation in patients with moderate ARDS (McNamee et al., 2021).

## Conclusion

Considering recent studies, RV failure in ARDS with its impact on outcome is now well recognized, as are its risk factors. Many studies suggest that to optimize respiratory settings it is essential to monitor RV function, while clinical impact of such a strategy on the outcome remains unclear. The RV protective approach should be prospectively evaluated in the future to improve the prognosis of the most seriously ill patients. ECMO could be part of this strategy in the most extreme situations.

## Data Availability Statement

The original contributions presented in the study are included in the article/supplementary material, further inquiries can be directed to the corresponding author.

## Author Contributions

MP, EJ, and AV-B wrote the manuscript. All authors contributed to the article and approved the submitted version.

## Conflict of Interest

AV-B is the recipient of a research grant from GSK. The remaining authors declare that the research was conducted in the absence of any commercial or financial relationships that could be construed as a potential conflict of interest.

## Publisher’s Note

All claims expressed in this article are solely those of the authors and do not necessarily represent those of their affiliated organizations, or those of the publisher, the editors and the reviewers. Any product that may be evaluated in this article, or claim that may be made by its manufacturer, is not guaranteed or endorsed by the publisher.
